# Development of hematopoietic syndrome mice model for localized radiation exposure

**DOI:** 10.1038/s41598-020-80075-w

**Published:** 2021-01-08

**Authors:** M. H. Yashavarddhan, Ajay Kumar Sharma, Pankaj Chaudhary, Sania Bajaj, Sukhvir Singh, Sandeep Kumar Shukla

**Affiliations:** 1 National Institute of Cancer Prevention & Research, Indian Council of Medical Research, Sector-39, Noida, Uttar Pradesh 201301 India; 2 Institute of Nuclear Medicine and Allied Sciences, Defence Research and Development Organisation, Brig. S K Mazumdar Marg, Timarpur, Delhi, 110054 India; 3 The Patrick G Johnston Centre for Cancer Research, Queen’s University Belfast, Belfast, UK

**Keywords:** Biological techniques, Biotechnology, Cell biology, Ecology, Molecular biology

## Abstract

Current models to study the hematopoietic syndrome largely rely on the uniform whole-body exposures. However, in the radio-nuclear accidents or terrorist events, exposure can be non-uniform. The data available on the non-uniform exposures is limited. Thus, we have developed a mice model for studying the hematopoietic syndrome in the non-uniform or partial body exposure scenarios using the localized cobalt^60^ gamma radiation exposure. Femur region of Strain ‘A’ male mice was exposed to doses ranging from 7 to 20 Gy. The 30 day survival assay showed 19 Gy as LD_100_ and 17 Gy as LD_50._ We measured an array of cytokines and important stem cell markers such as IFN-γ, IL-3, IL-6, GM-CSF, TNF-α, G-CSF, IL-1α, IL-1β, CD 34 and Sca 1. We found significant changes in IL-6, GM-CSF, TNF-α, G-CSF, and IL-1β levels compared to untreated groups and amplified levels of CD 34 and Sca 1 positive population in the irradiated mice compared to the untreated controls. Overall, we have developed a mouse model of the hematopoietic acute radiation syndrome that might be useful for understanding of the non-uniform body exposure scenarios. This may also be helpful in the screening of drugs intended for individuals suffering from radiation induced hematopoietic syndrome.

## Introduction

Historical radiation exposure incidents such as nuclear bombing over Japan during World War II, Chernobyl nuclear power plant disaster, Mayapuri radiation exposure in India, and Fleurus accident of Belgium indicate higher chances of partial body or non-uniform exposure^1–4^. Sometimes radiation over-exposure accidents during theranostic modalities may also result in heterogeneous, total or partial body exposures, which may lead to acute radiation syndrome with as low as 0.5 Gy dose of radiations resulting in ARS or acute radiation syndrome^5,6^. The signs and symptoms of ARS develop over months (sometimes within days) leading to systemic damage and mortality if not diagnosed and treated on time^7,8^. The ARS sub-syndrome, hematopoietic acute radiation syndrome (H-ARS) results from excessive damage to hematopoietic progenitor cells (HPCs) leading to heavy loss of blood cells^9–11^. It is also characterized by altered serum cytokines levels and the hematopoietic stem cell (HSCs) population^12,13^. However, H-ARS is responsive to medical countermeasures therefore, animal models which mimic this disease are of high importance for better understanding of the disease and for development of its countermeasures.

The current in-vivo models of H-ARS are based on whole-body exposure^14–18^. In last decade these models are extensively used for development of medical countermeasures (MCMs) against H-ARS. The whole body H-ARS models have been used to study the survival efficacy of potential radioprotectors and mitigators candidates, for dose optimization of candidate MCMs, polypharmacy of MCMs, and in pharmacokinetic/ pharmacodynamic studies^19–23^. Recently, animal models are studied for H-ARS treatment by the administration of cytokines and/or stem cells^24–26^. These models may not be sufficient to precisely simulate the risk of irradiation in scenarios involving facility accidents, dirty bomb explosion and radioisotope handling contamination accidents where exposures are mainly non-uniform or heterogeneous^7,27–31^. Thus, the importance of heterogeneous exposures that involves the exchange of cellular signalling molecules from irradiated to non-irradiated biological tissues such as abscopal effects may be underestimated by previously developed models, which can significantly complicate the biological outcomes of exposures^32^.

Therefore, the current study is broadly directed at the development of H-ARS mouse model using localized radiation exposure directly to bone marrow containing femur region. The bone marrow is specifically targeted as it is involved in blood cell formation and restoration of damaged hematopoietic organs (spleen and thymus) by differentiating stem cells which also contributes to make it the most radiosensitive tissue^33^. In the present study, radiation dose optimization was carried out considering lethality as an end-point by exposing both the femurs of mice. We also assessed the indirect radiation effects on spleen, thymus and blood using cell counting. Besides these studies, various growth factors and cytokines which play an important role in H-ARS were also measured. The observations of the current study affirm the better understanding of H-ARS by partial body exposure and the developed animal model could also be used for the efficacy and safety studies for development of MCMs.

## Materials and methods

### Chemicals and reagents

Sodium chloride (S3014), potassium chloride (P9541), sodium phosphate dibasic (S3264), potassium phosphate monobasic (P9791), potassium bicarbonate (12602), ammonium chloride (A9434), ethylene diamine tetra acetic acid di-sodium salt (E6635), bovine serum albumin (BSA) (5482), absolute ethanol (100983), methanol (34860) were purchased from Sigma–Aldrich, St. Louis, MO, United States. CD 34, Sca 1, and CBA Flex Set which contains IL-3 (558346), IL-6 (558301), TNFα (558299), IFN γ (562233), G-CSF (560152), GM-CSF (558347), IL-1α (560157), IL-1β (560232), Mouse/Rat Soluble Protein Master Buffer Kit (558266) were procured from BD Biosciences, United States. May-Grunwalds Stain (S039) and Giemsa Stain Solution (TCL083) were procured from Hi-Media India.

1 L 1X PBS (8 g of sodium chloride, 0.2 g of potassium chloride, 1.44 g of sodium phosphate dibasic, 0.25 g of potassium phosphate monobasic to 1 L at pH 7.4), 1 L 1X RBC lysis buffer (1 g of potassium bicarbonate, 8 g of ammonium chloride and 0.03 g of di-sodium EDTA), 1% BSA in PBS, 70% ethanol in PBS, and may-grunwald-giemsa stain mixed in 3:1 ratio were prepared in the laboratory.

### Animals and groupings

Pathogen free Strain ‘A’ male mice weighing 25.0–30.0 gm were received from Institute of Nuclear Medicine and Allied Science (INMAS) experimental animal facility at the age of 9 weeks, which is equivalent to young adult humans. Six mice per cage were housed under 25 ± 3 °C temperature and relative humidity of 30–70% in 12 h light/dark cycle with standard food and water. The study design strictly adhered to the guidelines approved by Institutional Animal Care and Use Committee (IACUC) of our institute, Institute of Nuclear Medicine and Allied Sciences (INMAS), Defence Research and Development Organization (DRDO), Delhi, India (Institute Animals Ethics Committee number: INM/IAEC/16/21).

After 1 week of acclimatization under ambient conditions, mice were grouped for the survival assay according to localized gamma radiation dose i.e. 7, 9, 10, 12, 15, 17 and 19 Gy. Eighteen animals were assigned in each group for this assay.

For all other parameters mice were divided into two groups with six animals in each. Group 1, consisted of untreated or sham irradiated animals. Group 2 was exposed to 15 Gy localized irradiation. Mice were sacrificed at different time points (1, 2, 4, 7, 10, 15, 20, 25, and 30 days) post treatment. Hematopoietic stem cell marker CD 34 and Sca1 were measured on 1, 4, 7, and 10th day post-irradiation.

### Irradiation

**Figure 1:**
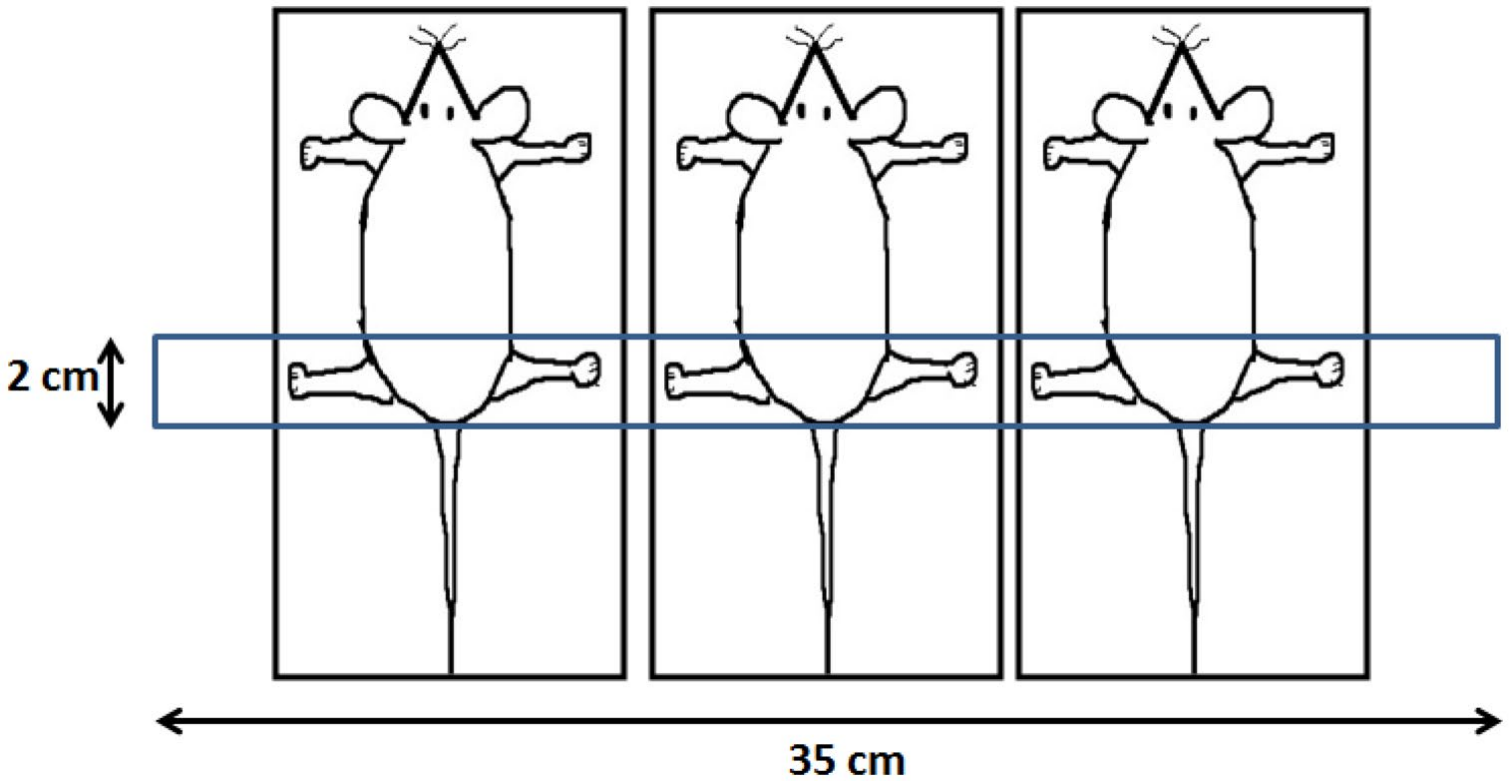
Representative image of animal exposure with γ-rays in a field size of 2 cm × 35 cm.

A Cobalt-60 teletherapy (Model: Bhabhatron-II, Panacea Medical Technologies Pvt Ltd, India) machine was used to irradiate the hinds of the three mice at a time (Fig. 1). The cobalt-60 beam was calibrated following IAEA TRS-398 protocol, with measurement done in water phantom (30 cm × 30 cm × 30 cm), for a field size 10 cm × 10 cm at source to surface distance (SSD) of 80 cm. A Farmer type, 0.6 cc volume, ionization chamber was utilised and the same was recently calibrated for absorbed dose to water (N_DW_) at Secondary Standard Dosimetry Laboratory (SSDL) of India. The measured absorbed dose rate (water, 10 cm × 10 cm, 80 SSD, Dmax) was 1.39 Gy/min.

The localized bone marrow irradiation was done using single Posteroanterior (PA) beam with 2 cm × 35 cm field size at 80 cm SSD opened through secondary collimators of the machine. The 5 mm thick acrylic re-strainers were used to immobilize each mouse which also provides the build-up thickness for the irradiation. The dose prescription point was the surface of the hinds i.e. 5 mm below the surface of the re-strainer. The whole irradiation platform was placed on 5 cm thick acrylic slab to provide sufficient back scatter and all the air gaps between the three mice were filled with tissue equivalent gel bolus or wet cotton to compensate for missing side scatter. The prescription doses were absorbed dose to water and not corrected for any tissue heterogeneity.

The absolute and relative dosimetry of collimated field (2 cm × 35 cm) were performed using pin point (0.04 cc volume) ionization chamber and EBT3 (ISP, Wayne, USA) Gafchromic dosimetry films in solid water phantom. The response of the pin point chamber and EBT3 films was calibrated against the SSDL calibrated ionization chamber in solid water phantom at the depth of 5 cm and field size 10 cm × 10 cm in the same beam quality (i.e. cobalt-60). The film scanning was performed using Epson 10000XL flatbed scanner as per the manufacturers scanning protocol. The beam profiles were obtained from the scanned films and the full width at half maximum (FWHM) in transverse direction was 1.78 cm for the opening of 2.0 cm. The dose uniformity was better than 4% in central 80% of the field. The absolute dose rate (dose to water, dmax, 2 cm × 35 cm, SSD 80 cm) measured at the centre of the field was 1 Gy/min. The overall uncertainty in dose calculations is 6.5% with enhancement or coverage factor (k = 2) at 95% confidence. The main body of the mice was covered with 15 mm lead to prevent any scatter radiation reaching at other body parts of the animal. The surface dose reaching at the shielded body parts of the mice was measured using 0.4 cc ionisation chamber. The measured mean surface dose was 0.024 ± 0.014% of the open field dose. This dose is negligible and considered to be acceptable.

### Survival assay in mice

For survival assay, 18 mice from each experimental group i.e. 7, 9, 10, 12, 15, 17 and 19 Gy, were irradiated at a fixed dose rate of 1 Gy/min. Groups were inspected daily for their morbidity and mortality status for a total duration of 30 days. All the surviving mice were euthanized humanely using intra-peritoneal injection (i.p.) with dexmedetomidine (0.3 mg/kg) in combination with ketamine (190 mg/kg) at the completion of the observation period.

### Validation of hematopoietic syndrome model

The model of hematopoietic syndrome was validated with localized radiation of 15 Gy as this was the maximum dose at which all the animals survived as observed in the survival results. Mice were euthanized using intra-peritoneal injection (i.p.) with dexmedetomidine (0.3 mg/kg) in combination with ketamine (190 mg/kg) at 1, 2, 4, 7, 10, 15, 20, 25, and 30th day time points. Further analysis such as organ weight, cell counting, and serum cytokines measurement assays were conducted on various organs such as femur bone, spleen, thymus, lungs, kidneys (L), kidneys (R), liver and heart and blood at above mentioned time points. Hematopoietic stem cell markers (CD 34 and Sca 1) were measured in bone marrow cells of mice at 1, 4, 7, and 10th day time points.

### Body and organ weights

The control and 15 Gy locally irradiated group animals were sacrificed at 1, 4, 7, 10, 15, 20, 25, and 30th day time points. The organs (spleen, thymus, lungs, kidneys (L), kidneys (R), liver and heart) were dissected out and weighed individually. Relative organ weight was calculated as the ratio between organ weight and body weight.

### Haematology

The blood samples were collected from untreated and 15 Gy localized radiation group at 1, 4, 7, 10, 15, 20, 25, and 30th day time points in EDTA vial by cardiac puncture. 20 µl of collected blood from each experimental animal was taken for haematology and analysed using Nihon Kohden Celltac α, Tokyo, Japan, a fully automatic 3 part haematology analyzer.

### Bone marrow cell counting

Total bone marrow cells were flushed out at 1, 4, 7, 10, 15, 20, 25 and 30th day points in 1 ml PBS from both femurs of the untreated mice and 15 Gy localized radiation group. The PBS containing cells was centrifuged at 2000 rpm for 8 min and the supernatant was discarded. The cells were washed with 1 ml PBS and the cell pellet was re-suspended in 1 ml of PBS. The re-suspended sample was diluted as required. The cells were then counted using improved Neubauer chamber under Olympus BX-63 microscope.

### Bone marrow smears study

Bone marrow cells collected at 1, 4, 7, 10, 15, 20, 25, and 30th day post-irradiation from different treatment groups were centrifuged at 2000 rpm for 8 min and re-suspended in 50 μl of Fetal Bovine Serum (FBS). A small drop of cells suspension was dropped on a clean microscopic slide and then smeared thinly over an area of 2–3 cm by pulling another glass slide held at a 45° angle. Cells were fixed with methanol and stained with may-grunwald-giemsa. Slides were then analysed using Olympus BX-63 microscope.

### Splenocytes and thymocytes counts

The organs excised at 1, 4, 7, 10, 15, 20, 25, and 30th day time-points were cleaned in chilled PBS. Using sterile-chilled frosted slides both the thymus and spleen were minced into a cell suspension and which was further centrifuged at 2000 rpm for 8 min. After centrifugation the supernatant was discarded. The RBCs were lysed using RBC lysis buffer and the cells were washed with 1 ml PBS. The cells were then counted using improved Neubauer chamber under Olympus BX-63 microscope.

### Measurement of hematopoietic stem cell marker CD 34 and Sca1

Bone marrow cells were isolated from both the femurs from various treatment groups at different time points. Cell were washed with chilled PBS and fixed with 70% chilled ethanol and stored overnight at – 20 °C. Following day, five million cells were blocked with 1% BSA-PBS and stained with CD 34 and Sca 1 cell-surface markers for 20 min at room temperature. 10,000 cells from each sample were analyzed using FACS LSR II (BD Biosciences, San Jose, CA) and results were visualized by the BD FACSDiva V7 software (BD Biosciences, San Jose, CA).

### Cytokines expression changes

Mice blood samples were collected in BD serum separation tube at different time-points post treatment (1, 4, 7, 10, 15, 25, and 30 days) via. cardiac puncture from different treatment groups. Samples were incubated at room temperature for 2 h and after incubation centrifuged at 6000*g* for 15 min. Serum was separated and stored at − 80 °C until analysis. The Levels of IL-3, IL-6, TNFα, IFN γ, G-CSF, GM-CSF, IL-1α,and IL-1β cytokines in mice serum were determined by using respective BD Cytometric Bead Array (CBA) Flex Set (BD Biosciences, USA) on dual laser flow analyzer (LSR-II, Becton Dickinson Biosciences, USA) according to manufacturer’s instructions. Results were analysed using FCAP Array V3 software (BD Biosciences, San Jose, CA).

### Statistical analysis

All values obtained in our results are represented as mean ± SD of three independent replicates. The 30 day survival data was plotted using Kaplan–Meier analysis. The difference between the experimental groups was evaluated by one-way analysis of variance, with Newman–Keuls multiple comparison test (V, 8.01; GraphPad Prism, San Diego, CA, USA). The comparisons were made among the untreated and 15 Gy locally irradiated groups for all experimental parameters except the survival study. A value of p < 0.05 is considered statistically significant. *p < 0.05, **p < 0.01, ***p < 0.001, ***p < 0.0001, ns = not significant (p > 0.05).

## Results

### Survival assay

**Figure 2:**
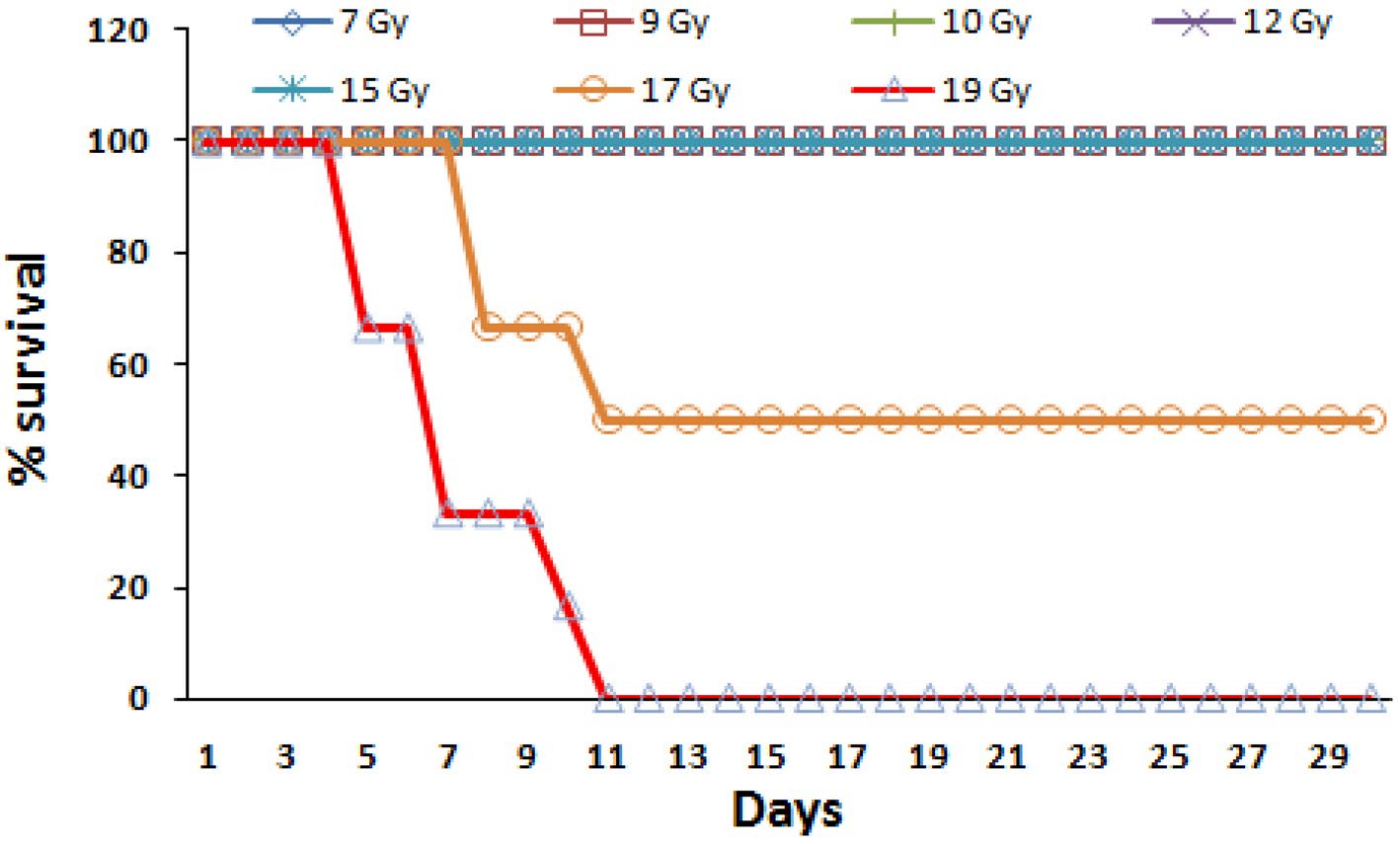
Effect of various doses of radiation (localized exposure to both femurs) on mice survival. All the animals survived in 7, 9, 10, 12, 15 Gy localized irradiation group with LD_50_ and LD_100_ as 17 and 19 Gy respectively. For survival assay, mice were randomly divided into seven groups of six mice in each group and observed for a period of 30 days for radiation induced morbidity and mortality. Survival experiment was independently repeated thrice. Data obtained was statistically analyzed and represented by a Kaplan–Meier survival curve.

After the localized irradiation of femurs in strain ‘A’ mice, we observed a gradual loss in the body weight, food and water intake and ruffled fur. 50% of the animals were found dead in case of 17 Gy dose and 100% of the animals died in case of 19 Gy dose within 11 days. In 15 Gy dose animals were in morbid state, however, none of the animals died in this group. Animals from all other lower than 15 Gy dose groups initially showed a loss of body weight which later recovered and within 30 days the weight of all the surviving mice was corresponding to untreated animals (Fig. 2).

### Haematology

**Figure 3:**
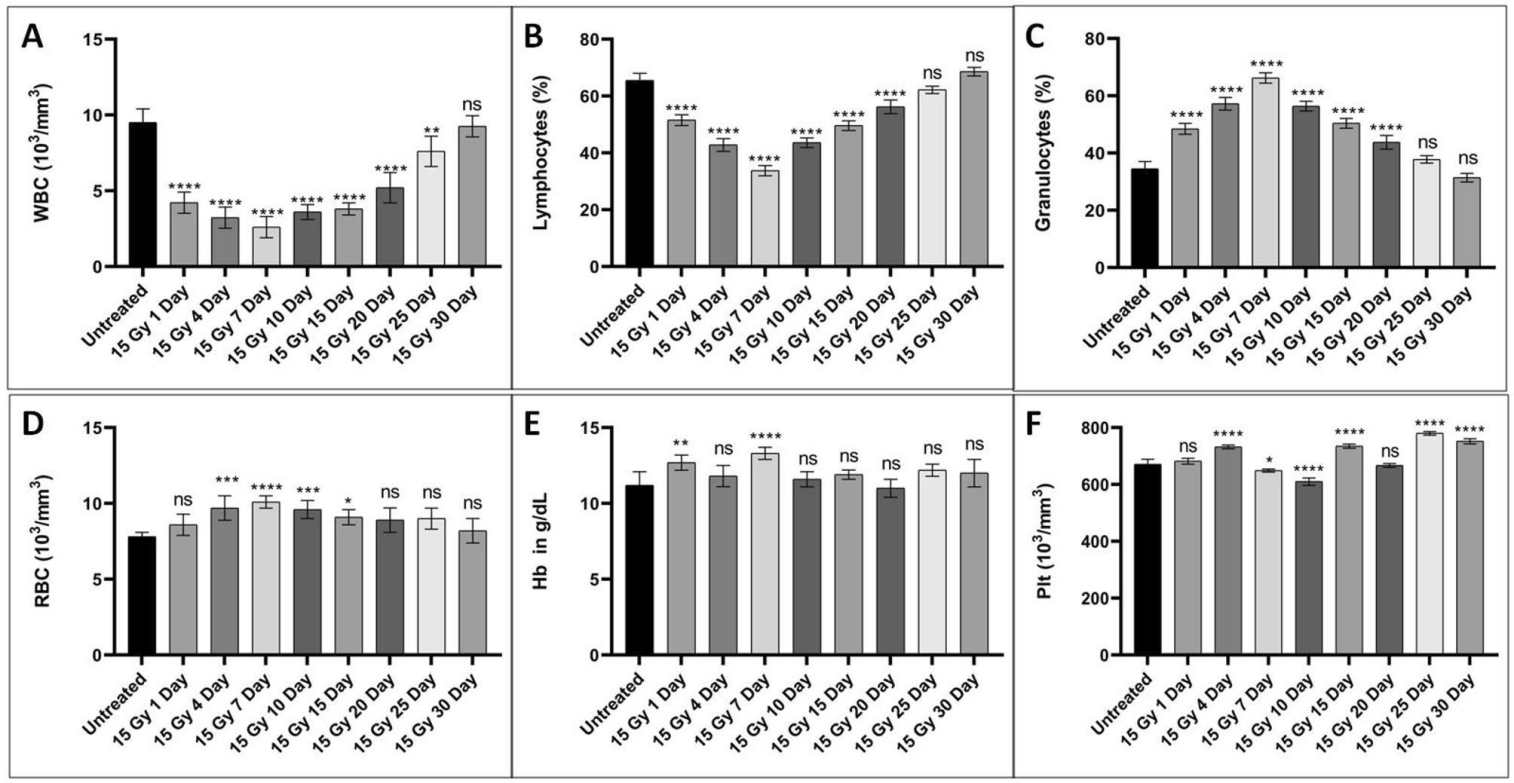
Cell counts in mice peripheral blood after 15 Gy localized radiation exposure at different time points. (**A**) Indicate WBC counts in the blood collected at various time points from mice. (**B**,**C**) represent lymphocytes and granulocytes percentage respectively. (**D**) Bars reflect RBC counts, (**E**) shows haemoglobin quantity in dL of blood, and (**F**) indicates the platelets counts at different time interval. Cells were counted using an automated haematology analyzer. The bars represent the mean ± SD of 6 animals. A value of p < 0.05 is considered statistically significant *p < 0.05, **p < 0.01, ***p < 0.001, ****p < 0.0001**,** ns = not significant (p > 0.05).

Mice in the different treatment groups were euthanized and blood collected by cardiac puncture at 1, 4, 7, 15, 20, 25, and 30th day post-irradiation. In this study we focused on the counting of total leucocytes (WBC), and the differential WBCs counts in terms of lymphocytes and granulocytes. We also analyzed the RBCs, haemoglobin and platelets (Fig. 3). In the untreated samples, the WBCs counts were 9.5 ± 0.9 million cells/ml and the percentage of lymphocytes and granulocytes were 65.5 ± 2.5% and 34.5 ± 2.5% respectively. A significant (p < 0.001) decrease in blood cell counts were observed upto 7 days when compared with untreated controls. After the day 7, the counts started increasing and normalized at 30th day post irradiation to those of untreated controls. The percentage of lymphocytes *vs* granulocytes cells found altered after radiation exposure. Maximum alteration in terms of percentage observed on 10th day post irradiation and the values were comparable to control by the 30th day post irradiation (Fig. 3).The untreated samples have 7.8 ± 0.3 10^3^/mm^3^ RBCs, 11.2 ± 0.9 g/dL haemoglobin, and 671 ± 17.7 10^3^/mm^3^ platelets counts. Significant (p < 0.05) changes in RBCs counts were observed on 4, 7, 10 and 15th day post irradiation when compared with the controls (Fig. 3). No particular patterns in the changes were observed in case of haemoglobin quantity and platelets counts (Fig. 3). The haematology results showed decrease in WBCs and increase in RBCs of mice exposed to 15 Gy localized radiation. The 15 Gy localized exposure also affected the lymphocytes and granulocytes percentage. However, no particular pattern was followed after radiation exposure in case of haemoglobin and platelet.

### Organ weight

Mice in the different treatment groups were euthanized at various time points on days 0, 1, 4, 7, 10, 15, 20, 25, and 30th after 15 Gy localised radiation to both femur as described in irradiation section. Spleen, thymus, lungs, liver, kidney (L), kidney (R), and heart w/o blood were isolated washed with normal saline and weighed. Relative organ weight was calculated as the ratio between organ weight and body weight. We observed significant changes (p < 0.001) only in case of spleen and thymus weight (Table 1). All other organs showed non-significant (p > 0.05) changes.

**Table 1:** Different organ weight of mice exposed to 15 Gy of localized radiation at various time points.

Organ	Weight (g)								
	Controls	1 Day	4 Day	7 Day	10 Day	15 Day	20 Day	25 Day	30 Day
Body weight	27.5 ± 1.5	26.5 ± 1.25	21.5 ± 2.5	18.2 ± 0.5	18.8 ± 0.7	20.0 ± 1.2	22.5 ± 0.75	23.5 ± 1.25	26.5 ± 2.2
Spleen	0.12 ± 0.01	0.08 ± 0.01	0.07 ± 0.02	0.03 ± 0.05	0.04 ± 0.01	0.04 ± 0.03	0.06 ± 0.03	0.09 ± 0.05	0.10 ± 0.04
Thymus	0.043 ± 0.002	0.024 ± 0.003	0.010 ± 0.002	0.004 ± 0.002	0.008 ± 0.002	0.015 ± 0.001	0.022 ± 0.002	0.038 ± 0.001	0.051 ± 0.001
Lungs	0.22 ± 0.022	0.20 ± 0.014	0.21 ± 0.012	0.19 ± 0.010	0.22 ± 0.017	0.23 ± 0.016	0.22 ± 0.016	0.23 ± 0.006	0.23 ± 0.005
Liver	1.9 ± 0.13	2.0 ± 0.12	2.07 ± 0.08	1.94 ± 0.13	1.98 ± 0.13	2.1 ± 0.17	2.04 ± 0.15	1.98 ± 0.12	1.95 ± 0.09
Kidney (L)	0.641 ± 0.02	0.64 ± 0.01	0.68 ± 0.05	0.66 ± 0.07	0.63 ± 0.06	0.6 ± 0.03	0.62 ± 0.04	0.66 ± 0.04	0.65 ± 0.07
Kidney (R)	0.628 ± 0.03	0.6 ± 0.07	0.598±	0.6 ± 0.1	0.64 ± 0.1	0.62 ± 0.4	0.63 ± 0.3	0.629 ± 0.5	0.618 ± 0.3
Heart w/o blood	0.15 ± 0.02	0.17 ± 0.01	0.157 ± 0.01	0.15 ± 0.1	0.155 ± 0.02	0.152 ± 0.03	0.151 ± 0.01	0.1620.01 ±	0.158 ± 0.04

### Total bone marrow counts and differential studies between nucleated and non-nucleated cell

**Figure 4:**
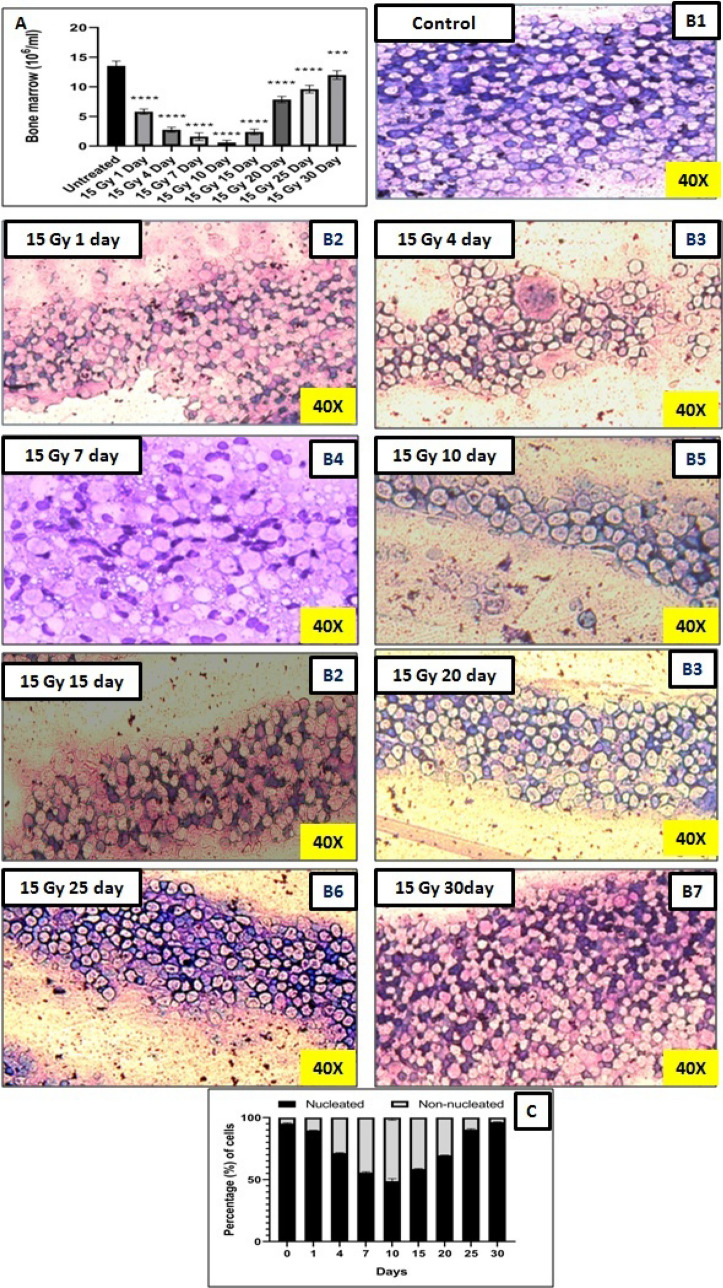
Represents the bone marrow counts and differential bone marrow counts in terms of nucleated and non-nucleated cells at different time points (**A**) Bone marrow counts of untreated and treated mice at various time intervals. (**B 1–7**) Representative images of bone marrow smear which shows nucleated and non-nucleated cells of untreated and treated samples at various time points. (**C**) The bars represent the nucleated and non-nucleated cells population post-irradiation. The bars represent the mean ± SD of 6 animals. A value of p < 0.05 is considered statistically significant *p < 0.05, **p < 0.01, ***p < 0.001, ****p < 0.0001, ns = not significant (p > 0.05).

Mice in the different treatment groups were euthanized and bone marrow cells were isolated by flushing the femurs with 1X PBS from untreated and treated groups on 1, 4, 7, 15, 20, 25, and 30th day post-irradiation and the cells were scored microscopically using Neubauer chamber. In the untreated group, the bone marrow count was 13.53 ± 0.83 million cells/ml. A significant (p < 0.0001) decrease in bone marrow cell count was recorded at all the time points. The linear decreasing trend in the cell counts persisted upto the day 10th post-irradiation and then an increase was observed. However, even after 30th day the counts are not comparable with the control groups. Cell counts were 5.80 ± 0.45, 2.74 ± 0.43, 1.59 ± 0.65, 0.60 ± 0.37, 2.38 ± 0.47, 7.84 ± 0.53, 9.60 ± 0.65, and 11.99 ± 0.74 million cells/ml at 1, 4, 7, 15, 20, 25, and 30th day time intervals respectively (Fig. 4A). 15 Gy localized radiation exposure resulted in reduction in cell number and alter nucleated and non-nucleated cell population. Post-radiation exposure resulted in an increase in non-nucleated cells with a decrease in number of nucleated cells (Fig. 4B,C).

### Total splenocyte and thymocytes counts after localized radiation exposure

**Figure 5:**
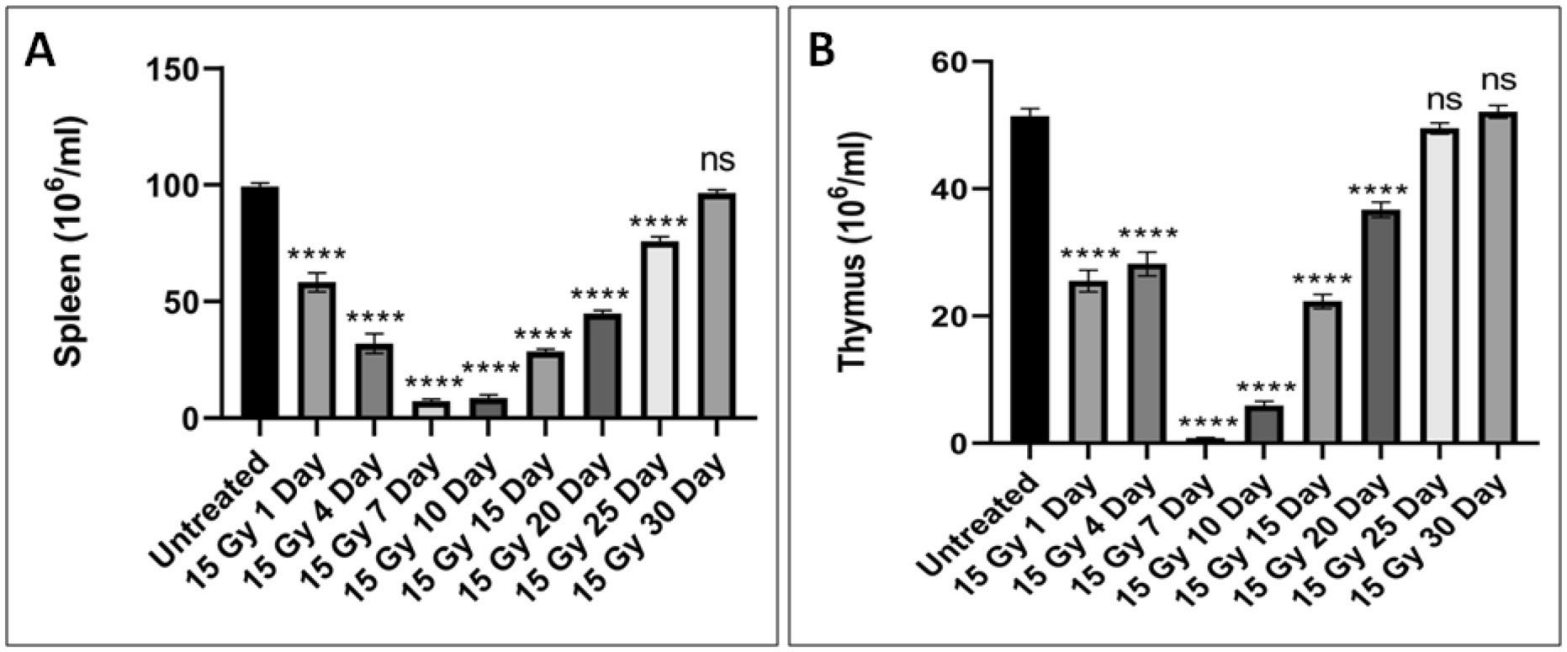
Effect of 15 Gy localized dose on the splenocyte and thymocyte counts in mice post-irradiation. (**A**) Splenocytes counts of untreated and treated mice at various time interval. (**B**) Thymocytes counts of untreated and treated mice at various time intervals. The bars represent the mean ± SD of 6 animals. A value of p < 0.05 is considered statistically significant *p < 0.05, **p < 0.01, ***p < 0.001, ****p < 0.0001**,** ns = not significant (p > 0.05).

Mice in various groups were euthanized to excise out the spleen and thymus. Both the spleen and thymus were minced on frosted slides to prepare a single cell suspension in PBS and the cells were counted using the Neubauer chamber. We observed that splenocyte and thymocyte counts reduced significantly after day 1 of irradiation (15 Gy localized exposures) and this linear reduction persisted upto the day 7. After the day 7, an increase in the counts was observed comparable to the untreated controls at day 30th post-irradiation as shown in Fig. 5. The 15 Gy localized radiation exposure to femur region resulted in severe damages to spleen and thymus.

### Effect of localized irradiation on the hematopoietic stem cell biomarkers expression

**Figure 6:**
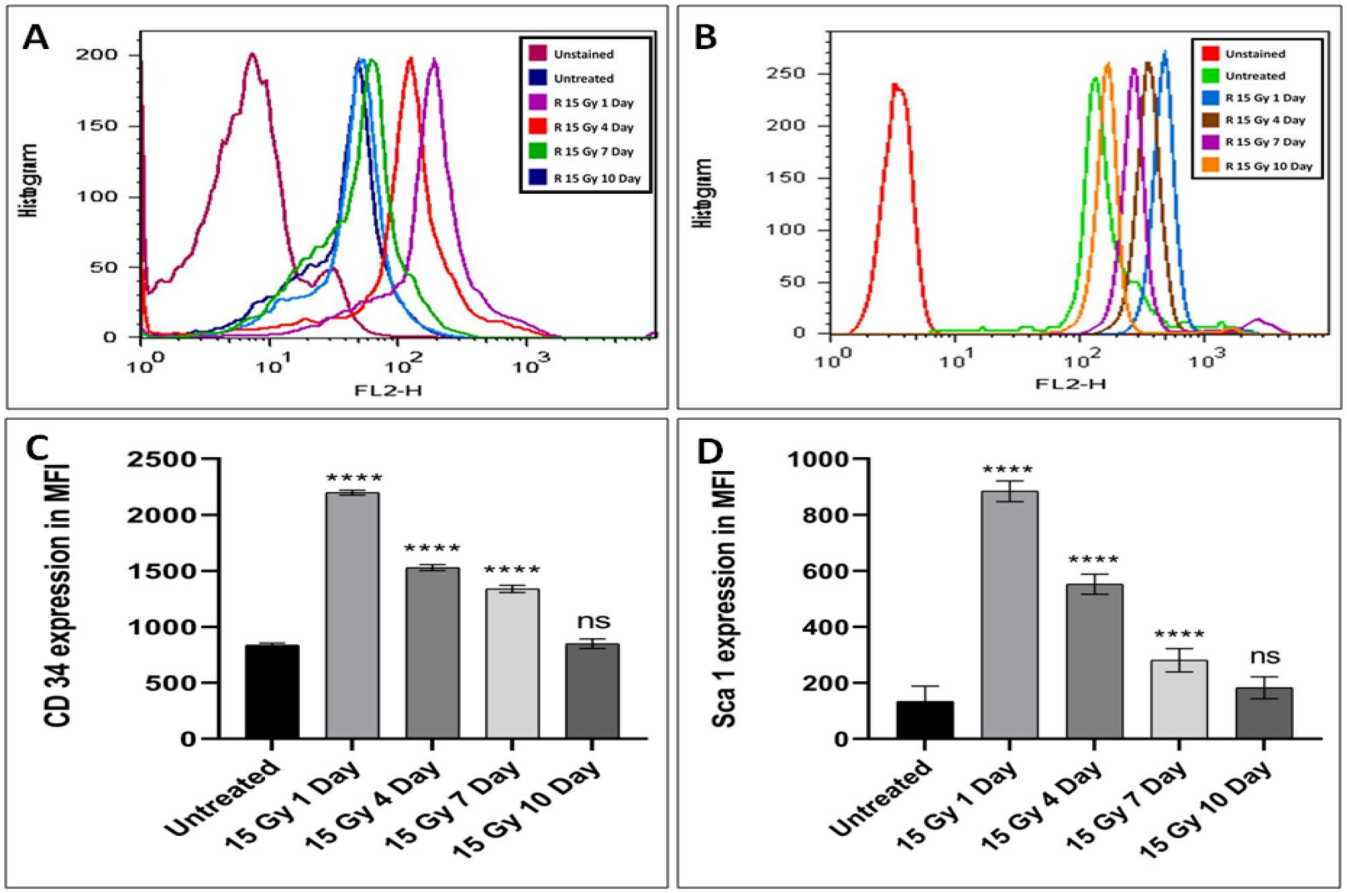
Hematopoietic stem cell marker expression in mice bone marrow cells after 15 Gy localized radiation exposure. (**A**,**B**) represents the flow cytometry data in the form of histograms of CD 34 and Sca 1 respectively. (**C**) Bars indicate CD 34 expression in mouse bone marrow at various time intervals. (**D**) Bars indicate Sca 1 expression in mouse bone marrow at various time intervals. The results were analyzed at BD-FACS LSR II using BD FACSDiva V7 program. The bars represent the mean ± SD of 6 animals. A value of p < 0.05 is considered statistically significant *p < 0.05, **p < 0.01, ***p < 0.001, ****p < 0.0001**,** ns = not significant (p > 0.05).

The bone marrow cells were isolated, washed and fixed with 70% ethanol. Cells were then counted and 1 million cells each were used for CD 34 and Sca 1 analysis using flow cytometry. We noticed a significant (p < 0.0001) increase in both the CD 34 and Sca1 cell surface marker expression up to 7th day and the Mean Fluorescent Intensity (MFI) was comparable to control at day 10 post-irradiation. The CD 34 and Sca 1 expression in terms of MFI in the untreated samples were 837 ± 18.07 and 134 ± 35.10 respectively. The post-irradiation values of CD 34 MFI were respectively 2200 ± 22.36, 1530 ± 26.48, 1340 ± 32.39, and 850 ± 41.76 on day 1, 4, 7, and 10 respectively. The post irradiation values of Sca 1 MFI were 885 ± 36.72, 281 ± 41.78, 183 ± 39.21, and 850 ± 41.76 on days 1, 4, 7, and 10 respectively (Fig. 6). Remarkable increase in CD 34 and Sca 1 expression observed upto a week upon 15 Gy local radiation exposures. These adult bone marrow cells have tremendous differentiation ability and restore various hematopoietic and other cell populations.

### Localized radiation induced changes in the cytokines levels in mouse serum samples

**Figure 7:**
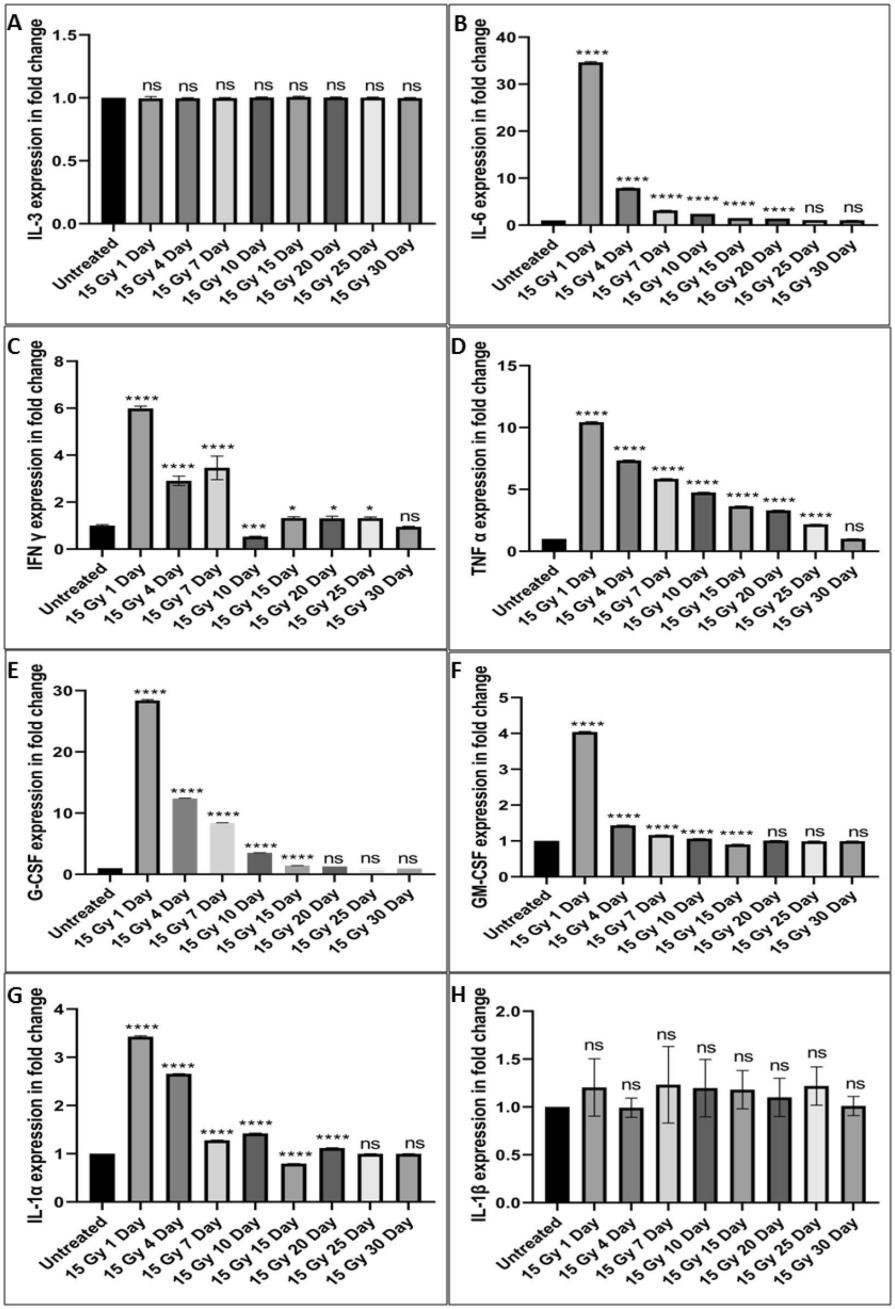
Changes in cytokines levels after localized 15 Gy exposure. (**A**–**H**) Bars indicate expression of IL-3, IL-6, G-CSF, GM-CSF, TNFα, IFNγ, IL-1α, and IL-1β. The results were analyzed at BD LSR II using BD FCAP Array V3 software. The bars represent the mean ± SD of 6 animals. A value of p < 0.05 is considered statistically significant *p < 0.05, **p < 0.01, ***p < 0.001, ****p < 0.0001, ns = not significant (p > 0.05).

At different time point serum levels of IL-3, IL-6, TNFα, IFN γ, G-CSF, GM-CSF, IL-1α, and IL-1β cytokines (pg/ml) were determined in the blood by flow cytometry using respective BD Cytometric Bead Array (CBA) Flex Set. Serum level of IL-3 and IL-1β remained unchanged in the irradiated group at all the time points when compared with untreated samples. On comparison with untreated controls IL-6, G-CSF, GM-CSF, TNFα, IFN γ and IL-1α values in the localized irradiated mice were found significantly up-regulated on the day 1 after irradiation (Fig. 7). All the values were comparable to untreated samples at 30 day post irradiation (Fig. 7).

## Discussion

Valid animal models according to US-FDA’s Animal Rule (AR) are imperative to address the type of exposure, the dose and time dependent incidences, as well as stringency of H-ARS. In addition, these H-ARS models can also be used for testing a medical counter measure (MCM) against H-ARS to demonstrate its efficacy, mechanisms of action, pharmacokinetics, pharmacodynamics and toxicity. Rodent models are applied for identifying potential MCMs and to study their *in-vivo* mechanisms of action. The literature includes several studies conducted over a decade, which defines the H-ARS small models against Total Body Irradiation (TBI)^9,15,18,20,34^. Similar TBI induced H-ARS studies are also reported in large animals such as mini pig and NHPs^35–38^. However, there are no contemporary studies that determine the local/partial exposure induced H-ARS in animal models.

In this study, we addressed development of a mouse model for hematopoietic syndrome of acute radiation syndrome by local/partial radiation exposure. The developed model can be useful in addressing two fundamental aspects related to hematopoietic syndrome: mechanistic studies of local radiation exposure induced hematopoietic syndrome and identification of potential MCMs for hematopoietic syndrome of acute radiation syndrome.

We performed localized radiation exposure to both femur of mice with 7, 9, 10, 12, 15, 17 and 19 Gy, and observed 17 Gy and 19 Gy the LD_50_ and LD_100_ dose respectively. Thus, 15 Gy radiation dose was chosen for development of H-ARS as doses above 15 Gy were lethal in mice. Further, we compared changes in blood components (WBC, lymphocytes, granulocytes, RBC, hemoglobin and platelets), weight and the counts of the internal organs. We observed the WBC count of mice decreased drastically after localized exposure to radiation and the number was comparable to control by 30th day post-irradiation. Similar changes are reported in various animal models (both small and large animals) as well as in humans^11,23,38–41^. Lymphocytes and granulocytes percentage were also affected in the blood after radiation exposure. We noticed a significant drop in lymphocytes percentage and an increase in the granulocytes percentage upto 7th day. This temporary reduction in the percentage of lymphocytes was observed because of the heightened susceptibility of the lymphocytes to radiation^23,42–44^. However, the temporary increase in granulocytes percentage was due to the pooling phenomenon, in which blood cells stored in the spleen leak into the peripheral vessels to compensate or rescue the cell loss^23,38,39,45^. Significant increase in RBC count was observed at day 4 and persisted upto 15th day post-irradiation. We did not observe any particular pattern in the increase or decrease in hemoglobin and platelet measurements. Our observation was in line with the previously observed results in case H-ARS of both human and animal model systems^40,46,47^. 15 Gy exposures led to marked loss in mice body weight along with hematopoietic organs weight (spleen and thymus) upto 10th day post-irradiation which was gradually restored by the 30th day post irradiation. This result corroborated with the cell counts of spleen and thymus which shows continuous loss in cell number in this group upto 10th day post irradiation and beyond that restoration process started with full recovery at 30th day post irradiation.

Several conventional radiobiology studies have shown the loss of body weight along with the loss in most of the organ weight during the first initial days post-irradiation in non-lethal to sub-lethal cases^48–51^. However, our result shows the damages only to hematopoietic system organ due to the local radiation exposure to bone marrow cells.

In the current study, we also observed the changes in the bone marrow cell counts along with the differential bone marrow cell population counts in the form of nucleated- and non-nucleated- cells, which confirmed radiation induced damage to the nucleated bone marrow cells. The large number of non-nucleated cells implies the presence of RBCs at later time points in radiation alone group which acts as an indicator of vascular damage. The bone marrow cell counting studies also corroborated a decline in the nucleated cell population in the bone marrow. Several studies in the past have also reported the observation of similar trend in the animal models in response to a very low dose of radiation^23,52–54^. Our results further support such observations.

Radiation exposure is known to induce early changes in cytokines expression to maintain various organ cellularity and inflammation^12,13,55,56^. Reports are also available which shows alteration in various pro- and anti- inflammatory cytokines, chemokines and growth factors found in case of radiotherapy treatment of various cancer patients^57,58^. In general, cytokine stimulation plays an imperative role in the survival, proliferation and differentiation^13,59^ of hematopoietic stem cells under various stressed conditions. The literature also showed that alteration in several kinds of cytokines led to dramatic increases in the hematopoietic stem cells population^60^. In case of whole body radiation exposure, it is evident that the expression of hematopoietic stem cells population increased significantly at early days^61,62^. However, there is no such information is available in case of partial body irradiation. The present study revealed that remarkable increase in CD 34 and Sca 1expression upto a week even after local radiation exposure of 15 Gy. Our finding corroborate with the finding of various available whole body exposure studies. The increased expression of two hematopoietic stem cells markers i.e. CD 34 and Sca1observed due to the alteration in various cytokines (IL-6, G-CSF, GM-CSF and TNFα) as shown in Fig. 7.

## Conclusion

The results of the current study demonstrate that 15 Gy local radiation exposures to femur region of mice shows significant alteration in organs of hematopoietic system which leads to development of radiation induced hematopoietic syndrome. Overall, we have developed a novel model system that will enable an un-paralleled approach for better understanding as well as enhancing the screening and validation capacity of MCMs against H-ARS.
