# Author Correction: Development of hematopoietic syndrome mice model for localized radiation exposure

**DOI:** 10.1038/s41598-022-17370-1

**Published:** 2022-08-02

**Authors:** M. H. Yashavarddhan, Ajay Kumar Sharma, Pankaj Chaudhary, Sania Bajaj, Sukhvir Singh, Sandeep Kumar Shukla

**Affiliations:** 1 National Institute of Cancer Prevention & Research, Indian Council of Medical Research, Sector‑39, Noida, Uttar Pradesh 201301 India; 2 Institute of Nuclear Medicine and Allied Sciences, Defence Research and Development Organisation, Brig. S K Mazumdar Marg, Timarpur, Delhi 110054 India; 3 The Patrick G Johnston Centre for Cancer Research, Queen’s University Belfast, Belfast, UK

Correction to: *Scientific Reports* 10.1038/s41598-020-80075-w, published online 08 January 2021

This Article contained an error in the ethical approval statement.

The text in the Materials and Methods, under the subheading *Animals and groupings*,

“The study design strictly adhered to the guidelines approved by Institutional Animal Care and Use Committee (IACUC) of our institute, Institute of Nuclear Medicine and Allied Sciences (INMAS), Defence Research and Development Organization (DRDO), Delhi, India (Institute Animals Ethics Committee number: INM/IAEC/2019/01)”

now reads

“The study design strictly adhered to the guidelines approved by Institutional Animal Care and Use Committee (IACUC) of our institute, Institute of Nuclear Medicine and Allied Sciences (INMAS), Defence Research and Development Organization (DRDO), Delhi, India (Institute Animals Ethics Committee number: INM/IAEC/16/21)”

Additionally, the Acknowledgements were also corrected,

“The authors sincerely thank the Director, INMAS for providing the necessary infrastructural support to accomplish this work. The authors are grateful to Dr. Manju Lata Gupta retired Scientist ‘G’ and former Project Director, RAKSHAK project for valuable guidance during her tenure. The authors acknowledge Dr. B. G. Roy for providing animals. Support from Mrs. Namita Kalra from INMAS and Dr. Vinay Gupta from BD Biosciences India in the flow-cytometry measurements is duly acknowledged.”

now reads

“The authors sincerely thank the Director, INMAS for providing the necessary infrastructural support to accomplish this work. The authors acknowledge Dr. B. G. Roy for providing animals. Support from Mrs. Namita Kalra from INMAS and Dr. Vinay Gupta from BD Biosciences India in the flow-cytometry measurements is duly acknowledged.”

The Article has been corrected.

